# Efficacy of icotinib in advanced lung squamous cell carcinoma

**DOI:** 10.1002/cam4.1736

**Published:** 2018-08-14

**Authors:** Shuai Liang, Yan Xu, Fenlai Tan, Lieming Ding, Yongbin Ma, Mengzhao Wang

**Affiliations:** ^1^ Division of Respiratory Medicine Peking Union Medical College Hospital Peking Union Medical College Chinese Academy of Medical Sciences Beijing China; ^2^ Zhejiang Betta Pharmaceuticals Hangzhou China

**Keywords:** adenocarcinoma, EGFR, EGFR‐TKIs, icotinib, squamous cell carcinoma

## Abstract

**Background:**

There are controversial data supporting the efficacy of epidermal growth factor receptor (EGFR) tyrosine kinase inhibitors (TKIs) in patients with advanced lung squamous cell carcinoma (SCC). In this study, the efficacy of icotinib in unselected and *EGFR*‐mutated patients with lung SCC was assessed.

**Methods:**

We retrospectively analyzed the survival time of unselected advanced lung SCC patients treated with icotinib for at least 5 months between June 2013 and June 2016, and selected appropriate *EGFR‐*mutated advanced lung ADC patients to have 1:1 ratio of propensity score matching with *EGFR*‐mutated advanced lung SCC patients, and matching factors were age, sex, clinical stage, Karnofsky performance status (KPS), smoking history, *EGFR* mutation type, and treatment lines.

**Results:**

A total of 487 unselected advanced lung SCC patients were available for analysis of icotinib treatment efficacy. The progression‐free survival (PFS) was 13.0 months (95% CI 12.2‐13.8), the overall survival (OS) was 16.0 months (95% CI 14.7‐17.3), and the objective response rate (ORR) was 41.3%. After propensity score matching, 78 *EGFR*‐mutated lung SCC and 78 *EGFR*‐mutated lung ADC patients were selected and compared. Although no statistical difference was found, ADC patients were associated with a longer PFS (15.8 months vs 12.7 months, *P *=* *0.275) and OS (24.2 months vs 18.5 months, *P *=* *0.150), and a better ORR (59.0% vs 48.7%, *P *=* *0.199) than compared with SCC patients when treated with icotinib.

**Conclusion:**

Icotinib has a modest therapeutic effect in patients with advanced lung SCC, especially for the population with *EGFR* mutations.

## 1 INTRODUCTION

Lung cancer is the leading cause of cancer‐related death worldwide.^1^ Non‐small‐cell lung cancer (NSCLC) constitutes approximately 80% of all lung cancers, and squamous cell carcinoma (SCC) is one of the major subtypes of NSCLC which accounts for approximately 20% to 30% of NSCLC.^2^ There are only a few treatment options for patients with lung SCC except chemotherapy. In recent decades, molecular targeted therapy has demonstrated clinical efficacy in cancer patients, such as epidermal growth factor receptor tyrosine kinase inhibitors (EGFR‐TKIs) for advanced NSCLC patients with *EGFR* mutations. EGFR‐TKIs had been proven to offer prolonged progression‐free survival (PFS) and better life quality than chemotherapy in advanced NSCLC patients with *EGFR* mutations in many clinical trials,^3^, ^4^, ^5^, ^6^, ^7^, ^8^, ^9^, ^10^ in which most of the patients were adenocarcinoma. However, the efficacy of EGFR‐TKIs in patients with lung SCC is limited, even in SCC patients with *EGFR* mutations.

*EGFR* mutation testing was an essential part of standard care for lung cancer. Several societies have issued guidelines and consensus statements regarding *EGFR* mutation testing in patients with lung SCC. According to the American Society of Clinical Oncology (ASCO), none of the patients with NSCLC should be excluded from having the *EGFR* genetic testing performed if the patient is being considered for first‐line therapy with an EGFR‐TKI and the decision is physician‐driven.^11^ In Europe, the consensus of the European Society for Medical Oncology (ESMO) suggests that *EGFR* mutation testing should be performed in patients who are never/former light smokers and in patients with nonsquamous cell carcinoma.^12^ The consensus guideline from the College of American Pathologist (CAP), International Association for the Study of Lung Cancer (IASLC), and Association for Molecular Pathology (AMP) suggests *EGFR* mutation testing in lung ADC, in tumors where an ADC component cannot be excluded, and in cases, whose clinical criteria are unusual.^13^ The National Comprehensive Cancer Network (NCCN) guideline adopts the idea and suggests the consideration of *EGFR* mutation testing in lung SCC especially in never smokers, small biopsy specimens, or mixed histology.^14^ In summary, ASCO recommends *EGFR* mutation testing in all patients with SCC when EGFR‐TKIs are considered, but ESMO/ACP/IASLC/AMP/NCCN suggests it only in some specific conditions.

In recent years, several prospective and retrospective studies have demonstrated that the frequency of *EGFR* mutations in patients with SCC was 3.9%‐17.2%, which was higher than expected.^15^, ^16^, ^17^ However, the efficacy of EGFR‐TKIs in *EGFR*‐mutated lung SCC is still controversial, and the tumor responses in SCC are much lower than ADC after EGFR‐TKIs treatment. Shukuya et al^18^ found the ORR in *EGFR*‐mutated lung SCC (n = 27) and ADC (n = 199) with gefitinib was 30% and 66%, respectively (*P *<* *0.001). Wu et al^19^ found the objective response rate (ORR) in *EGFR*‐mutated nonadenocarcinoma (n = 9) and ADC (n = 161) with gefitinib or erlotinib was 22.2% and 69.6%, respectively (*P *=* *0.003).

Icotinib, an orally administered EGFR‐TKI with high selectivity, has been used widely in China. In a Phase 3 randomized head‐to‐head trial (ICOGEN),^20^ icotinib was clinical equivalent to gefitinib in patients with NSCLC. The efficacy of icotinib for patients with SCC is not well known.

In this study, we decided to investigate the efficacy of icotinib in both unselected and *EGFR*‐mutated advanced lung SCC population

## 2 PATIENTS AND METHODS

### 2.1 SCC patients

Advanced unselected or *EGFR*‐mutated lung SCC patients treated with icotinib were retrospectively selected from expand access program (EAP) database of Betta Pharmaceuticals. The patients were from 230 lung cancer research centers between June 2013 and June 2016. The last follow‐up date was 1 April 2017. Baseline clinical characteristics including age, gender, smoking history, tumor histology, clinical stage, Karnofsky performance status (KPS), *EGFR* mutation status, and treatment lines were collected.

The inclusion criteria were pathologically confirmed locally advanced stage IIIB or metastatic stage IV SCC of the lung after at least 5 months treatment of icotinib before charity period, because patients were from EAP database. The exclusion criteria were as follows: (a) icotinib used as adjuvant therapy; (b) icotinib combined with chemotherapy; and (c) data were incomplete. The institutional ethnic commitment board of the Peking Union Medical College Hospital approved the study. All patients provided written informed consent before participation in the charity project.

### 2.2 Matching adenocarcinoma patients

There were 289 *EGFR*‐mutated lung adenocarcinoma patients from EAP database of Betta Pharmaceuticals were selected to have 1:1 ratio of propensity score matching with *EGFR*‐mutated lung SCC patients. The propensity scores, which were calculated from the logistic regression models, included the following variables: age, gender, clinical stage, KPS, smoking history, *EGFR* mutation type, and treatment lines. Through the matching procedure for propensity scores, the *EGFR*‐mutated SCC and *EGFR*‐mutated ADC groups showed similar distributions of propensity scores, indicating that the differences in covariates between the two groups were minimized. We matched propensity scores one by one using nearest neighbor methods, no replacement, and 0.03 clipper width. Finally, we matched 78 patients from *EGFR*‐mutated SCC group and 78 patients from *EGFR*‐mutated ADC group.

### 2.3 Test method for *EGFR* mutations

Mutations in the tyrosine kinase domain of *EGFR* were identified using the amplification refractory mutation system (ARMS). DNA was extracted from patients’ fresh tissue or paraffin‐embedded tissue. Not all patients with lung SCC were included in the *EGFR* mutation analysis.

### 2.4 Clinical assessments

Patients received 125 mg oral icotinib three times per day, a treatment cycle is 28 days until intolerable toxicity disease progression or death. According to EAP program, first‐time tumor imaging and routine laboratory test were performed 4 weeks after therapy, repeated every 8 weeks. The objective tumor responses were evaluated according to the Response Evaluation Criteria in Solid Tumors (RECIST 1.1).^21^ Objective tumor responses included complete response (CR), partial response (PR), stable disease (SD), and progressive disease (PD). Disease control rate (DCR) was defined as the addition of objective response and stabilization. The PFS was calculated from the date of initiation of icotinib therapy to the date of tumor progression or any cause of death. The duration of overall survival (OS) was calculated from the date of initiation of icotinib therapy to the date of death.

### 2.5 Statistical methods

Demographic and clinical data are expressed as medians with ranges for continuous variables, and categorical variables are expressed as the means of absolute and percentage numbers. The PFS and OS are expressed as median values with two‐sided 95% confidence intervals (CIs) and were analyzed with the Kaplan‐Meier method. Log‐rank test was used to compare the difference between groups. For multivariate analysis, Cox regression was done to select significant prognostic variables for survival, of which age, gender, clinical stage, KPS, smoking history, and tumor response were analyzed as factors. Statistical significance was defined as *P *<* *0.05. SPSS software, version 23 (SPSS Inc. Chicago, IL, USA) and GraphPad Prism 7.00 were used for all statistical analyses.

## 3 RESULTS

### 3.1 Patient characteristics

**Figure 1:**
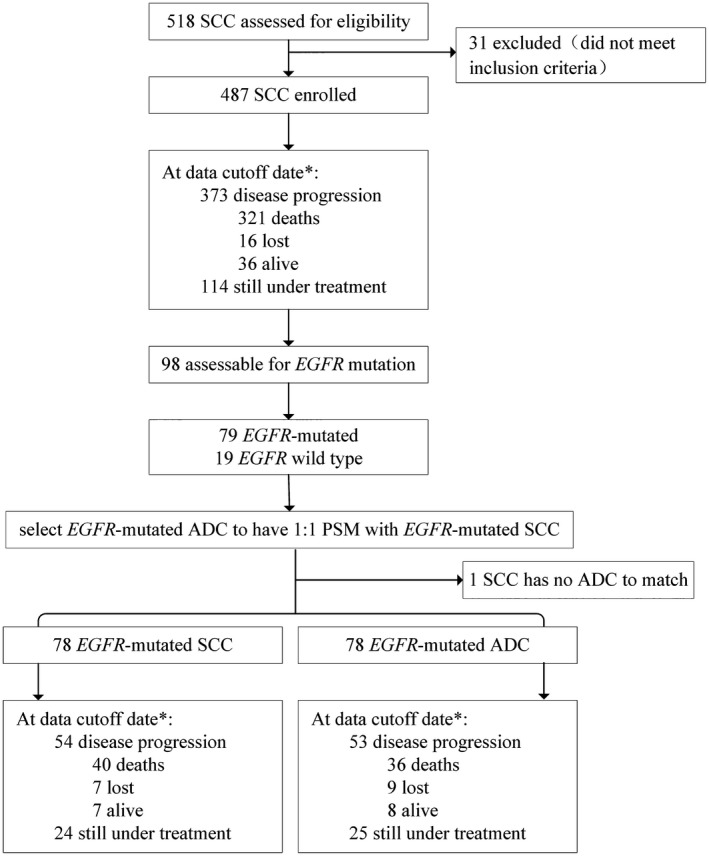
Flow diagram of patients studied. *Data cutoff date was April 1, 2017

Overall, 518 unselected patients with advanced lung SCC were treated with icotinib from June 2013 to June 2016 in EAP database of Betta Pharmaceuticals, of which 31 did not meet the inclusion criteria and excluded, leaving 487 patients with lung SCC for analysis. *EGFR* mutation status was tested in 98 of 487 patients with lung SCC (20.1%) in our study, which was not random, and there were 79 SCC patients *EGFR* mutation positive. The most common types of *EGFR* mutations were exon 19 deletion (36 patients) and exon 21 L858R (26 patients), and other mutation types were exon 18 (2 patients), exon 20 (1 patient), exon 20,21 (1 patient), exon21 L861Q (1 patient), exon 21 L858R+T790M (1 patient), and positive (11 patients). A total of 78 ADC patients with *EGFR* mutations were selected to compare with *EGFR*‐mutated SCC patients. One SCC patient with *EGFR* mutations was not matched because of old age, poor performance status, and early clinical stage. A flowchart is shown in Figure 1. The characteristics (age, gender, clinical stage, KPS, smoking history, *EGFR* mutation type, and treatment lines) of all patients were well balanced among groups and are summarized in Table 1.

**Table 1:** Patients demographic data and baseline characteristics

Characteristics	Before matching	After matching		
	Unselected SCC (n = 487)	*EGFR*‐mutated SCC (n = 78)	*EGFR*‐mutated ADC (n = 78)	*P*
Age (y)				
Median (range)	64 (28‐91)	63 (32‐83)	64 (47‐85)	0.422
<65 y	249 (51.1%)	44 (56.4%)	39 (50.0%)	
≥65 y	238 (48.9%)	34 (43.6%)	39 (50.0%)	
Gender				
Male	347 (71.3%)	45 (57.7%)	46 (59.0%)	0.871
Female	140 (28.7%)	33 (42.3%)	32 (41.0%)	
Clinical stage				
IIIB	126 (25.9%)	24 (30.8%)	27 (34.6%)	0.609
IV	361 (74.1%)	54 (69.2%)	51 (65.4%)	
KPS				
60‐80	30 (6.2%)	2 (2.6%)	3 (3.8%)	1.000
≥80	457 (93.8%)	76 (97.4%)	75 (96.2%)	
Smoking history				
Nonsmokers	195 (40.0%)	58 (74.4%)	58 (74.4%)	1.000
Smokers	261 (53.6%)	20 (25.6%)	20 (25.6%)	
Unknown	31 (6.4%)	0 (0%)	0 (%)	
*EGFR* mutation status				
Mutated	79 (80.6%)	78 (100%)	78 (100%)	0.262
*19 del*	36 (45.6%)	35 (44.9%)	28 (35.9%)	
*L858R*	26 (32.9%)	26 (33.3%)	36 (46.2%)	
Others^a^	17 (21.5%)	17 (21.8%)	14 (17.9%)	
Wild type	19 (19.4%)	0 (0%)	0 (%)	
Treatment lines				
First line	30 (6.2%)	10 (12.8%)	12 (15.4%)	0.792
Second line	32 (6.6%)	8 (10.3%)	6 (7.7%)	
Third line or more	4 (0.8%)	0 (0%)	0 (%)	
Unknown	421 (86.4%)	60 (76.9%)	60 (76.9%)	

ADC, adenocarcinoma; *EGFR*, epidermal growth factor receptor; SCC, squamous cell carcinoma.

*P* value: compare *EGFR*‐mutated SCC and *EGFR‐*mutated ADC patients.

a Other mutation types: *exon18/exon20/exon20,21/exon21 L861Q/T790M+exon 21 L858R*/positive.

### 3.2 Efficacy

**Figure 2:**
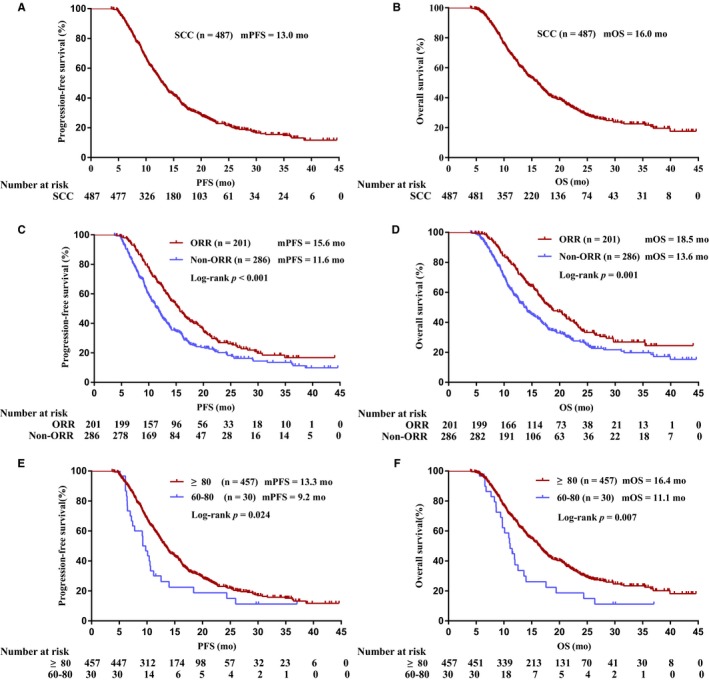
Kaplan‐Meier analysis of EGFR unselected lung SCC (A, B) progression‐free survival (PFS) and overall survival (OS) of unselected lung SCC; (C, D) PFS and OS of unselected lung SCC tumor response; (E, F) PFS and OS of unselected lung SCC KPS

**Figure 3:**
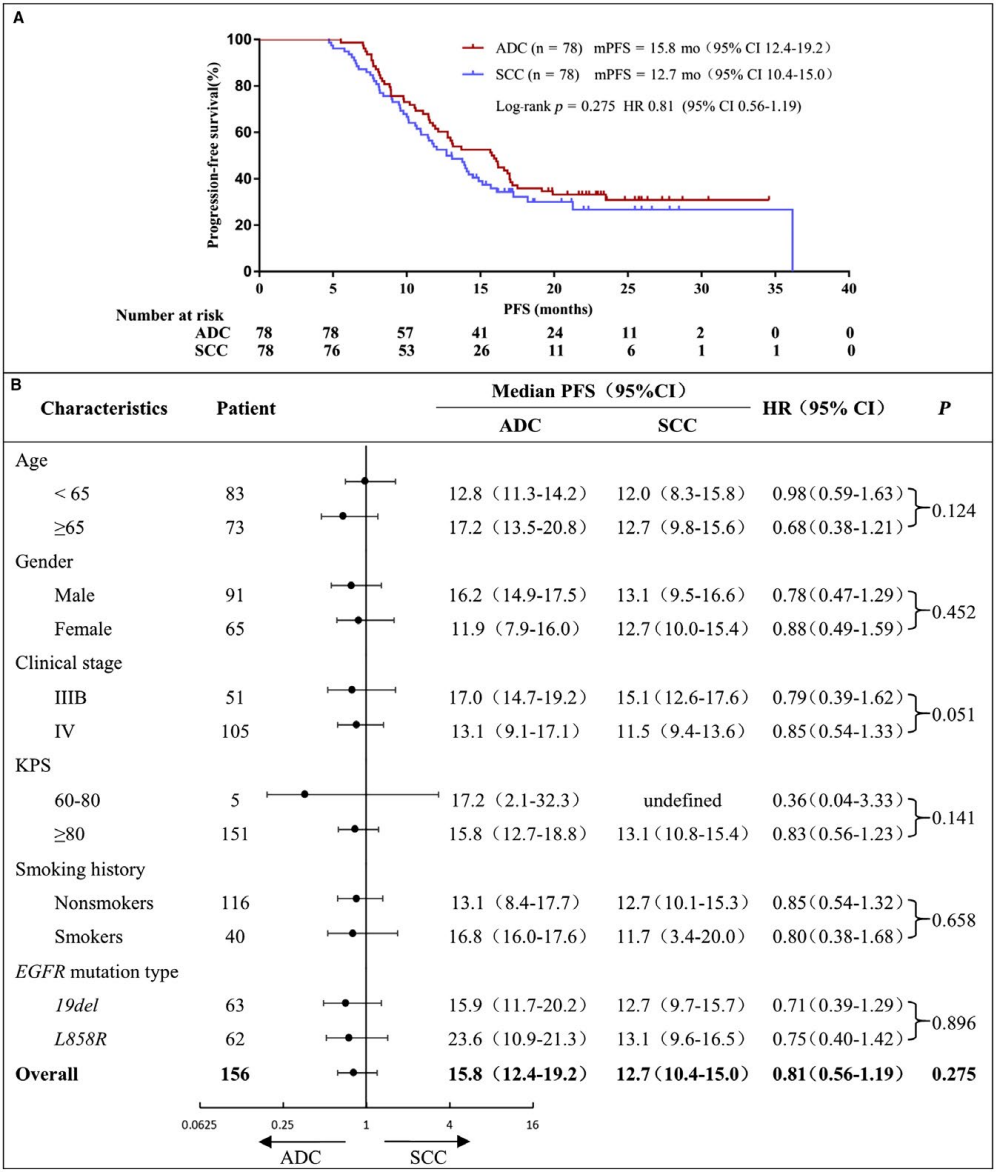
EGFR‐mutated lung SCC and ADC progression‐free survival (A) EGFR‐mutated lung SCC and ADC PFS curve. (B) PFS subgroup analysis by independent review

**Figure 4:**
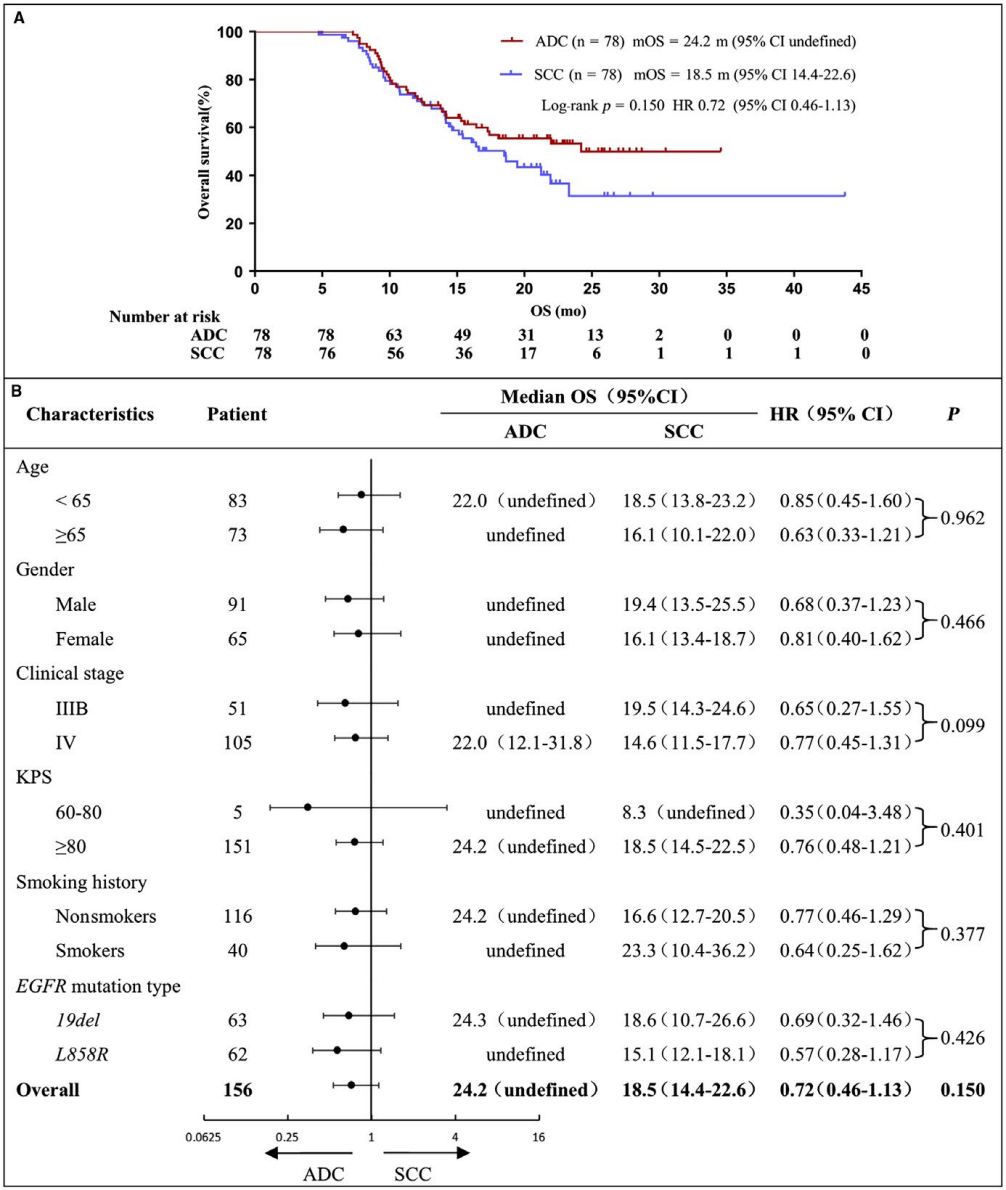
EGFR‐mutated lung SCC and ADC overall survival (A) EGFR‐mutated lung SCC and ADC OS curve. (B) OS subgroup analysis by independent review

The PFS for unselected lung SCC patients (n = 487) was 13.0 months (95% CI 12.2‐13.8), and OS was 16.0 months (95% CI 14.7‐17.3) (Figure 2A,B). Univariate analysis of unselected lung SCC patients PFS showed that patients with better KPS score and objective tumor response to icotinib had significant longer PFS (Figure 2C,E, Table 2), but in multivariate analysis, only objective tumor response had significant lower HR (Table 2). Both univariate analysis and multivariate analysis of unselected lung SCC patients OS demonstrated that better KPS score and objective tumor response to icotinib had significant better OS (Figure 2D,F; Table 2). Among *EGFR*‐mutated SCC (n = 78) and matching ADC (n = 78) patients, no significant difference in PFS and OS was found between the two groups (Figures 3A and 4A), although PFS and OS were slightly better in matching ADC patients than those in *EGFR*‐mutated SCC across subgroups such as age, gender, clinical stage, KPS, smoking history, and *EGFR* mutation type.

**Table 2:** Univariate and multivariate survival analysis for unselected lung SCC patients

Factor	Category	PFS				OS			
		Univariate		Multivariate		Univariate		Multivariate	
		HR (95% CI)	*P*	HR (95% CI)	*P*	HR (95% CI)	*P*	HR (95% CI)	*P*
Age	<65	0.94 (0.77‐1.15)	0.534	0.99 (0.80‐1.23)	0.955	0.82 (0.66‐1.02)	0.072	0.87 (0.69‐1.11)	0.259
	≥65								
Gender	Male	1.2 (0.96‐1.49)	0.115	1.17 (0.91‐1.50)	0.220	1.30 (1.03‐1.64)	0.039	1.20 (0.92‐1.57)	0.186
	Female								
Clinical stage	IIIB	0.85 (0.68‐1.07)	0.187	0.84 (0.66‐1.08)	0.165	0.88 (0.69‐1.12)	0.306	0.88 (0.68‐1.15)	0.344
	IV								
KPS	60‐80	1.57 (0.97‐2.56)	0.024	1.50 (0.98‐2.30)	0.063	1.74 (1.03‐2.92)	0.007	1.69 (1.09‐2.60)	0.018
	≥80								
Smoking history	Nonsmokers	0.96 (0.77‐1.19)	0.698	1.03 (0.82‐1.30)	0.789	0.86 (0.68‐1.09)	0.217	0.94 (0.73‐1.21)	0.625
	Smokers								
Tumor response	ORR	0.68 (0.56‐0.83)	<0.001	0.68 (0.55‐0.85)	0.001	0.68 (0.55‐0.85)	0.001	0.70 (0.55‐0.88)	0.003
	Non‐ORR								

Among the 487 unselected lung SCC patients, 21 achieved CR, 180 achieved PR, 285 had SD, and 1 had PD. The ORR was 41.3% (201/487), and DCR was 99.8% (486/487). Among the 78 *EGFR*‐mutated SCC patients, 1 had CR, 37 achieved PR, and 40 had SD; among 78 *EGFR*‐mutated ADC patients, 4 achieved CR, 42 achieved PR, and 32 had SD. There was no significant difference in ORR between *EGFR*‐mutated SCC and ADC (48.7% vs 59.0%, *P *=* *0.199).

The incidence of adverse events of icotinib was low in all groups, and the most common adverse events were rash, diarrhea, and raised transaminase (Table 3).

**Table 3:** Most common adverse events

	Unselected SCC (n = 487)	*EGFR*‐mutated SCC (n = 78)	*EGFR*‐mutated ADC (n = 78)
Rash	17 (3.5%)	1 (1.3%)	3 (3.8%)
Diarrhea	7 (1.4%)	2 (2.6%)	0 (0%)
Raised transaminase	12 (2.5%)	1 (1.3%)	0 (0%)

## 4 DISCUSSION

*EGFR* mutation rate was low in lung SCC, and data of efficacy of EGFR‐TKIs for patients with lung SCC are limited. Some studies have argued that response to *EGFR* targeted therapies in SCC is contributed to pathological mis‐classification,^22^ and it is also increasingly being recognized that different mutation testing systems have different sensitivity variations for detection of *EGFR* mutations.^23^ In BR.21and SATURN clinical trials, subgroup analysis showed that treatment with EGFR‐TKIs was effective in patients with SCC.^24^, ^25^ A meta‐analysis demonstrated that EGFR‐TKIs prolonged PFS and OS (*P *=* *0.004, *P *=* *0.04) compared with placebo in unselected patients with advanced lung SCC.^26^ But more trials reported that EGFR‐TKIs were less effective in patients with SCC, even in *EGFR*‐mutated SCC patients. Hata et al^27^ found that the ORR was 9.7%, DCR was 43.9%, median PFS was 2.2 months (95% CI 1.0‐2.8), and median OS was 11.0 months (95% CI 5.7‐15.7) in unselected lung SCC (n = 41) treated with erlotinib. Tseng et al^28^ found the ORR was 17.4%, DCR was 27.2%, median PFS was 1.7 months (95% CI 1.4‐2.0), and median OS was 4.4 months (95% CI 2.8‐7.1) in unselected lung SCC (n = 92) treated with erlotinib. In our study, the ORR and DCR in unselected lung SCC (n = 487) were 41.3% and 99.8%, and the median PFS and OS were 13.0 months (95% CI 12.2‐13.8) and 16.0 months (95% CI 14.7‐17.3), respectively. The favorable efficacy of EGFR‐TKIs in our study should be considered in the context that these patients with SCC had nonprogressive disease after 5‐month treatment of icotinib, which enriched the responsive patients.

The results in the present study showed that SCC patients with objective responses had better PFS and OS benefits than those without responses, suggesting that patients with lung SCC have a better tumor response to EGFR‐TKIs would have a better prognosis. Better PFS and OS benefits were also seen in unselected lung SCC patients with a KPS ≥80 compared with those with a KPS 60‐80. Performance status is an independent predictive factor of icotinib treatment outcome in unselected advanced lung SCC patients. This may provide a trend for clinician to choose EGFR‐TKIs treatment in patients with advanced lung SCC.

ADC patients with sensitizing *EGFR* mutations may survival about 30 months. However, controversial efficacy of EGFR‐TKIs was seen in *EGFR*‐mutated SCC patients. The OPTIMAL trial demonstrated that erlotinib was associated with a better PFS benefit for patients with *EGFR* mutations than standard chemotherapy,^7^ irrespective of histologic type, whereas there were only 10 nonadenocarcinoma patients enrolled in the erlotinib group. In the pooled analysis of Shukuya et al,^18^ the median PFS in *EGFR*‐mutated SCC (n = 27) and ADC (n = 199) with gefitinib was 3.1 months vs 9.4 months (*P *=* *0.0001), and the ORR was 30% vs 66%, respectively (*P *<* *0.001). In the pooled analysis of Wu et al,^19^ the median OS in *EGFR*‐mutated nonadenocarcinoma (n = 9) and ADC (n = 161) with gefitinib or erlotinib was 2.3 months vs 18.1 months (*P *<* *0.001), and the ORR was 22.2% vs 69.6%, respectively (*P *=* *0.003). A retrospective matched‐pair case‐control study^29^ found *EGFR*‐mutated SCC (n = 44) and ADC (n = 44) patients with EGFR‐TKIs had similar ORR (43.2% vs 54.5%, *P *=* *0.290), but patients with SCC had lower DCR (71.3% vs 100%, *P *=* *0.001), significant shorter median PFS (5.1 vs 13.0 months, *P *=* *0.000), and median OS (17.2 vs 23.6 months, *P *=* *0.027). In summary, benefits of EGFR‐TKIs in *EGFR*‐mutated SCC patients are inferior to *EGFR*‐mutated ADC patients; however, unmatched *EGFR*‐mutated SCC and ADC patients may lead to bias, and the sample size of *EGFR*‐mutated SCC patients was very small.

In this study, we collected 78 *EGFR*‐mutated SCC patients and matched with ADC patients to compare the efficacy of EGFR‐TKIs, and the results showed that median PFS in *EGFR*‐mutated SCC and ADC patients treated with icotinib was 12.7 months vs 15.8 months, median OS was 18.5 months vs 24.2 months, and the ORR was 48.7% vs 59.0%. No significant difference was detected between the two groups in PFS or OS.

In recent years, there are several molecularly targeted agents, and immunotherapies have provided a new level of optimism for patients with lung SCC. Anti‐*EGFR* monoclonal antibodies (necitumumab^30^ and cetuximab^31^, ^32^, ^33^) in combination with standard chemotherapy significantly improved lung SCC patients’ survival time with an acceptable safety profile. The immune‐checkpoint inhibitors nivolumab^34^ and pembrolizumab^35^ have demonstrated durable tumor responses and encouraging survival improvements vs standard cytotoxic agents. The anti‐VEGFR2 antibody ramucirumab has been approved in combination with docetaxel for the second‐line treatment of NSCLC, including lung SCC, based on the Phase III REVEL trial.^36^ The ErbB‐family blocker afatinib has demonstrated clinical activity in patients with lung SCC.^10^, ^37^, ^38^ Afatinib significantly improved the PFS, OS, and DCR vs erlotinib in the LUX‐Lung 8 trial,^39^ leading to its approval for locally advanced or metastatic lung SCC who had progressed after platinum‐based chemotherapy. The future for the treatment of lung SCC is increasingly promising, and we look forward to further developments in the coming years.

The results of the present study should be interpreted with the consideration of several limitations. The major limitation of this study was its retrospective nature, which had selection bias to a certain degree. Second, the small sample size in *EGFR‐*mutated SCC and ADC patients would affect the statistical analysis. Third, the present study could not obtain immunohistochemical results of all patients with SCC; therefore, we could not distinguish poorly differentiated adenocarcinoma. Some experts hold the view that some *EGFR‐*mutated SCC patients may also have a mixed ADC histology, and the sensitivity of EGFR‐TKIs in these patients might depend on the proportion of *EGFR*‐mutated ADC components in the whole tumor.^40^, ^41^ World Health Organization recommends immunohistochemistry not only for small biopsies/cytology, but also for resected specimens in certain settings such as solid ADC, nonkeratinization SCC, which guides the treatment.^42^ Furthermore, since the retrospective nature, the incidence of adverse events during the medication was lower than the actual situation. Prospective study with large sample was needed to over limitations mentioned above.

In conclusion, icotinib has some effects in unselected and *EGFR*‐mutated SCC patients as in ADC patients, who had received at least 5 months of icotinib treatment. Icotinib should be considered as a potential treatment option for this patient population, and *EGFR* mutation test should be recommended in all patients with SCC.

## ACKNOWLEDGMENTS

We acknowledge the Zhejiang Betta Pharmaceuticals at Hangzhou for providing medical records of study patients.

## CONFLICT OF INTEREST

The authors declare no conflict of interest.
